# Acute hyperlipidemia has transient effects on large-scale bone regeneration in male mice

**DOI:** 10.1038/s41598-024-76992-9

**Published:** 2024-10-27

**Authors:** Luciana Yamamoto de Almeida, Catharine Dietrich, Olivier Duverger, Janice S. Lee

**Affiliations:** Craniofacial Anomalies and Regeneration Section, National Institute of Dental and Craniofacial Research (NIDCR), National Institutes of Health (NIH), Bethesda, MD 20892 USA

**Keywords:** Developmental biology, Structural biology, Diseases, Medical research

## Abstract

Excessive dietary fat intake increases plasma lipid levels and has been associated with reduced bone mineral density (BMD) and increased risk of osteoporotic fracture, especially in older postmenopausal women. The objective of this study was to investigate whether there are sex-related differences in lipid metabolism that could have an impact on large-scale bone regeneration. Because ribs provide a unique exception as the only bones capable of completely regenerating large-scale defects, we used a rib resection mouse model in which human features are recapitulated. After 10 days of exposure to a low-fat diet or high-fat diet (HFD), we performed large-scale rib resection surgeries on male and female mice (6–7 weeks old) with deletion of the low-density lipoprotein (LDL) receptor (Ldlr^−/−^) and age- and sex-matched wild-type (WT) mice were used as controls. Plasma analysis showed that short-term exposure to HFD significantly increases total cholesterol, LDL cholesterol, and triglycerides levels in Ldlr^−/−^ mice but not in WT, with no differences between males and females. However, under HFD, callus bone volume was significantly reduced exclusively in male Ldlr^−/−^ mice when compared to WT, although these differences were no longer apparent by 21 days after resection. Regardless of diet or genotype, BMD of regenerated ribs did not differ significantly between groups, although male mice typically had lower average BMD values. Together, these results suggest that short-term hyperlipidemia has transient effects on large-scale bone regeneration exclusively in male mice.

**Supplementary Information:**

The online version contains supplementary material available at 10.1038/s41598-024-76992-9.

## Introduction

The ability of bones to repair spontaneously is limited to fractures and minimal tissue loss^1^, while the correction of large-scale bone defects (≥ 1 cm), also known as critical size defects^2^, require extensive surgical intervention with bone-grafting or bone substitutes, associated with a high risk of infection^3^. Therefore, despite advances in the fields of tissue engineering and regenerative medicine, the repair of large-scale bone defects remains a clinical challenge. From a clinical perspective, ribs are an excellent source of osteochondral grafting material and frequently used in many clinical settings of reconstructive surgical procedures, particularly for the craniofacial region^4^. However, unlike most mammalian bones, ribs have a unique spontaneous capacity for complete regeneration even when a large portion is removed, as previously observed in postoperative visits of patients who had undergone rib resection surgery for reconstruction of skeletal defects^5^ and experimental studies in mice^6–8^. This is the reason why rib defects were used as an experimental model for large-scale bone regeneration in the current study. However, limited knowledge has been described about the molecular mechanisms related to its remarkable intrinsic regenerative capacity, as well as the factors that could potentially influence this process. In this regard, robust lines of investigation are recently uncovering signaling pathways that are activated in skeletal stem and progenitor cells required for proper osteochondrogenic differentiation of the ribs^6,8^. Among them, it has been demonstrated that Hedgehog signaling activity is critical for osteochondrogenic tissue formation during large-scale rib regeneration^8^ and, at the same time, this pathway is reciprocally regulated by intracellular cholesterol biosynthesis in chondrocytes of long bones^9^. This suggests that there is positive feedback between cholesterol synthesis and chondrocyte differentiation and proliferation. However, environmental factors, including exposure to a high-fat diet (HFD), and sex-related differences in lipid metabolism have not yet been determined to influence the rib regeneration process.

Lipid homeostasis is beneficial for the organization and activity of signaling proteins in the plasma membrane^10^ and plays a fundamental role during skeletal development^11,12^. However, inborn errors of cholesterol synthesis cause malformation syndromes, including skeletal deformities^11,12^. Furthermore, pharmacological inhibition of cholesterol biosynthesis during embryogenesis induces skeletal defects^13^ and suppresses longitudinal growth of long bones^14^. Conversely, hyperlipidemia, which consists of elevated levels of lipids in the blood, including triglycerides, total cholesterol, low-density lipoprotein (LDL) cholesterol, and free fatty acids (FFA) is largely associated with an increased risk of atherosclerosis and cardiovascular disease^15^, and also involved in the development of osteoarthritis^16^ and osteoporosis^17^, especially in postmenopausal women, as a result of decreased estrogen levels^18–20^. Experimental hyperlipidemia can be induced in LDL receptor-deficient mice (Ldlr^−/−^) and blood lipid levels are further increased when fed diets containing high levels of fat and cholesterol. This model has been widely used to mimic human hyperlipidemia to understand its effects in different scenarios, including skeletal development and disease^21^. Mechanistically, Ldlr deficiency has been shown to decrease osteoclast formation and increase bone mass as a result of impaired cell-cell fusion of preosteoclasts^22^. In contrast, HFD induces bone mass loss in Ldlr^−/−^ mice, and this effect is at least in part associated with altered expression of *Runx2* (key transcription factor of osteoblast differentiation) and TRAP (resistant acid phosphatase), suggesting that HFD-fed mutant mice exhibit impaired osteoblast differentiation and increased osteoclastic function^23^. These studies were performed only in male mice and analysis of bone structure in the absence of Ldlr did not include the response to bone injury.

In the context of bone repair, Pirih et al.^24^ demonstrated that large-scale bone regeneration was significantly reduced in hyperlipidemic male mice fed a HFD, irrespective of the mice’s genetic background (wild-type/WT—Ldlr^+/+^ or Ldlr^−/−^) using a cranial bone defect mouse model in which spontaneous bone regeneration occurs, but only partially. However, the effects of HFD on the unique ability of ribs to completely regenerate their large defects, as well as metabolic differences related to sex that may affect bone repair, have not yet been investigated. Therefore, in the present study, we examined whether sex differences in lipid metabolism and exposure to a HFD would potentially reduce regenerated rib volume after surgical resection and its bone mineral density (BMD) in young mice at the peak of their regenerative capabilities. Understanding the sex- and diet-related differences that influence large-scale bone regeneration could provide insights into new ways to repair other bones and tissues. We consider that the results will provide new information that may help guide treatment decisions for large-scale bone defects in patients with hyperlipidemia.

## Materials and methods

### Animals

Female and male homozygous knockout mice lacking the low-density lipoprotein (LDL) receptor gene (Ldlr^−/−^, strain: B6.129S7-Ldlrtm1Her/J, background C57BL/6J) at 4–5 weeks of age and their wild-type (WT) age-matched controls (Ldlr^+/+^, strain: C57BL/6J) were obtained from Jackson Laboratory (Bar Harbor, ME). The age factor is generally considered as a potential confounding factor when examining phenotypic changes in health and disease, therefore, in the present study, age-related effects on bone regeneration capability and lipid metabolism were prevented by exclusively using young mice. All mice studied from each strain and sex were randomly divided into two dietary groups for a period of 10 days before rib resection surgery and maintained the diet until 21 days post-resection (dpr). Animals from each strain and sex were fed a high-fat diet (HFD) (Teklad Custom Research Diet TD.88137; 15.2% kcal protein, 42.7% kcal carbohydrates, 42.0% kcal fat, and 0.15% cholesterol added: 0.05% from fat source), while the remaining animals were kept on the standard low-fat diet (LFD) (LabDiet 5001, 28.7% kcal protein, 13.4% kcal fat and 57.9% kcal carbohydrates). Animals were housed up to 5 per cage for a 7-day acclimation period after being shipped by the vendor and before being allocated to dietary groups. After establishing the dietary group, each mouse was kept isolated in a cage until the end of the experiment (21 dpr), allowed free access to water and specific diet ad libitum, and were kept on 12 h light cycle. Male and female mice were used in this study to ensure the representation of any sex-related differences in the bone regenerative process. All experimental procedures were approved by the NIH/NIDCR Animal Care and Use Committee (Protocol number: #21-1081) and performed in accordance with relevant guidelines and regulations. This study was conducted in accordance with the ARRIVE guidelines (https://arriveguidelines.org).

### Measurement of plasma lipid levels

Blood was collected from the facial vein of mice (100 µL/mouse) at baseline and 0 dpr. Blood samples were centrifuged at 4 °C for 10 min at ~ 12,000*g* to obtain plasma. Then, the plasma samples were immediately aliquoted and stored at − 80 °C until the experiment was conducted. Plasma levels of LDL cholesterol were measured using enzyme-linked immunosorbent assay kits from Crystal Chem Inc. following the manufacturers’ instructions. Plasma levels of total triglycerides, total cholesterol, and free fatty acids (FFA) were all analyzed at the Mouse Metabolism Core Laboratory, National Institute of Diabetes and Digestive and Kidney Diseases (NIDDK/NIH). The volume of plasma required per sample for each assay is 3 µL.

### Murine rib resection surgeries

During the entire surgical procedure (~ 30–40 min), mice were induced and maintained under anesthesia by inhalation of the volatile anesthetic isoflurane (2% concentration). Large-scale rib resection surgeries were performed on 6–7 weeks old male and female WT and Ldlr^−/−^ mice as previously described^6,7^, and a 3 mm portion of bone was removed. The 8th or 9th ribs were selected to be excised from the right side of the rib cage of each mouse to ensure a minimally invasive surgical access that provides a large-scale resection due to their anatomical length, resulting in discontinued bone space, avoiding costochondral joint and cartilage. After rib resection, the proximal and distal parts of a rib remain stable and do not require the use of rigid fixators, since the ribs are not weight-bearing bones and, in addition, they are also surrounded by the periosteum and intercostal muscle groups that keep them in place. Sutures were placed to aid in the healing process, keeping the tissues around the discontinued bone space together. Pain management included pre-surgical injection of sustained-release buprenorphine (1 mg/kg) and topical application of bupivacaine (8 mg/kg) on the surgical site.

### In vivo µCT scanning

For in vivo µCT analysis, anesthetized mice (under continuous administration of isoflurane 2%) were scanned using the Quantum GX MicroCT (PerkinElmer, Inc. Waltham, MA) at the Mouse Imaging Facility (National Institute of Neurological Disorders and Stroke - NINDS/NIH) over 2, 7, 14, and 21 dpr. The X-ray source was set to a current of 88 µA, voltage of 90 kV. The field of view (FOV) was set to 36 mm × 36 mm, and voxel size was 72 μm.

### Ex vivo high-resolution µCT scanning

At 21 dpr, the mice were euthanized via CO_2_ inhalation followed by cervical dislocation. Dissection was performed to remove the rib cages and the samples were fixed for 48 h in 4% paraformaldehyde in 1× PBS, rotating at 4°C. After fixation, the regenerated rib and the unresected contralateral rib were excised and loaded in a 19 mm tube filled with 70% EtOH for ex vivo high resolution µCT. Scans were performed using a µCT 50 specimen scanner (Scanco Medical AG, Bassersdorf, Switzerland) with 70kVp X-ray source voltage, 85 µA beam intensity/beam current, 6 W power, collecting 2,000 projections in each rotation at 900 ms integration time for a 360-degree scan with a 0.18-degree rotation. The image resolution was 10 μm.

### µCT image analysis and quantification

All µCT scans (in vivo and ex vivo) were uploaded as DICOM format files into AnalyzePro 14.0 software (AnalyzeDirect, Overland Park, KS, USA) and calibrated for density using a hydroxyapatite (HA) phantom as a reference (in vivo: QRM, Moehrendorf, Germany, #QRM-MicroCT-HA D10, containing a range of known density values: 0, 50, 200, 800, 1200 [mg HA/cm^3^]; ex vivo: Scanco Medical AG, #K34-01-23, density values: 0, 200, 400, 600, 800 [mg HA/cm^3^]). After scanning the dissected ribs, the three-dimensional (3D) reconstructed images were created, and the regenerated bone volume (BV) and bone mineral density (BMD) were measured using AnalyzePro software (AnalyzeDirect, Overland Park, KS, USA). All surgeries were performed to consistently remove a 3 mm bone segment in the middle part of the rib, but the diameter and, consequently, the BV of each bone removed differed slightly between the resected ribs. Moreover, because the mice involved in this study are skeletally immature, for the analysis of in vivo µCT scans, after uploading the DICOM files into the Analyze Pro software, normalization must be performed to assess the amount of bone regeneration filling the discontinued bone space over physiological bone formation in growing mice at the proximal and distal ends of the resected rib. In this sense, to estimate the BV removed versus regenerated BV overtime, mice were scanned in a stage when the resected rib does not show any sign of bone regeneration (2 dpr), and at stages when the discontinued bone space start showing progressive bone regeneration (7 dpr, 14 dpr, and 21 dpr). Taking this into consideration, target ribs, either recently resected or in a stage when bone regeneration is detected by µCT analysis, were segmented in the software analysis from their adjacent bone ends (vertebral column and costal cartilage) to exclusively obtain complete BV measurements of the target rib and exclude other anatomically closely connected bone tissues. The BV of the respective contralateral rib was also measured in the same way and used as a reference for the total BV, as it was not resected. At 2 dpr, the total BV value of the target rib was normalized by subtracting it from the BV of the respective contralateral rib to obtain the estimated amount of removed bone. Since the discontinued bone space start showing progressive bone regeneration at 7 dpr, 14 dpr, and 21 dpr, the same calculation was performed, and the resulting values were consistently progressively decreasing overtime. Finally, each resulting normalized values obtained at 7 dpr, 14 dpr, and 21 dpr were subtracted by the normalized value obtained at 2 dpr to obtain the amount of bone volume that was regenerated. For analysis of ex vivo high resolution µCT scans, a region of interest (ROI) was determined using the middle portion of each repair callus and its respective unresected contralateral rib, and 200 slices from each sample were selected for analysis.

### Statistical analysis

Data are expressed as means ± standard deviation (SD) for the indicated number of observations. Shapiro-Wilk test was used to determine normality. Depending on the data distribution, one-tailed unpaired Student’s t test or Mann-Whitney test was used for comparisons in which there were two groups; two-way analysis of variance (ANOVA) followed by Fisher’s LSD test was applied for analyses in which there were three or more comparisons being made. A p value of less than 0.05 was considered statistically significant. Prism 7 (GraphPad Software) was used for all statistical analysis.

## Results

### Plasma lipid levels are exacerbated in Ldlr^−/−^ mice under high-fat diet regardless of sex

**Fig. 1:**
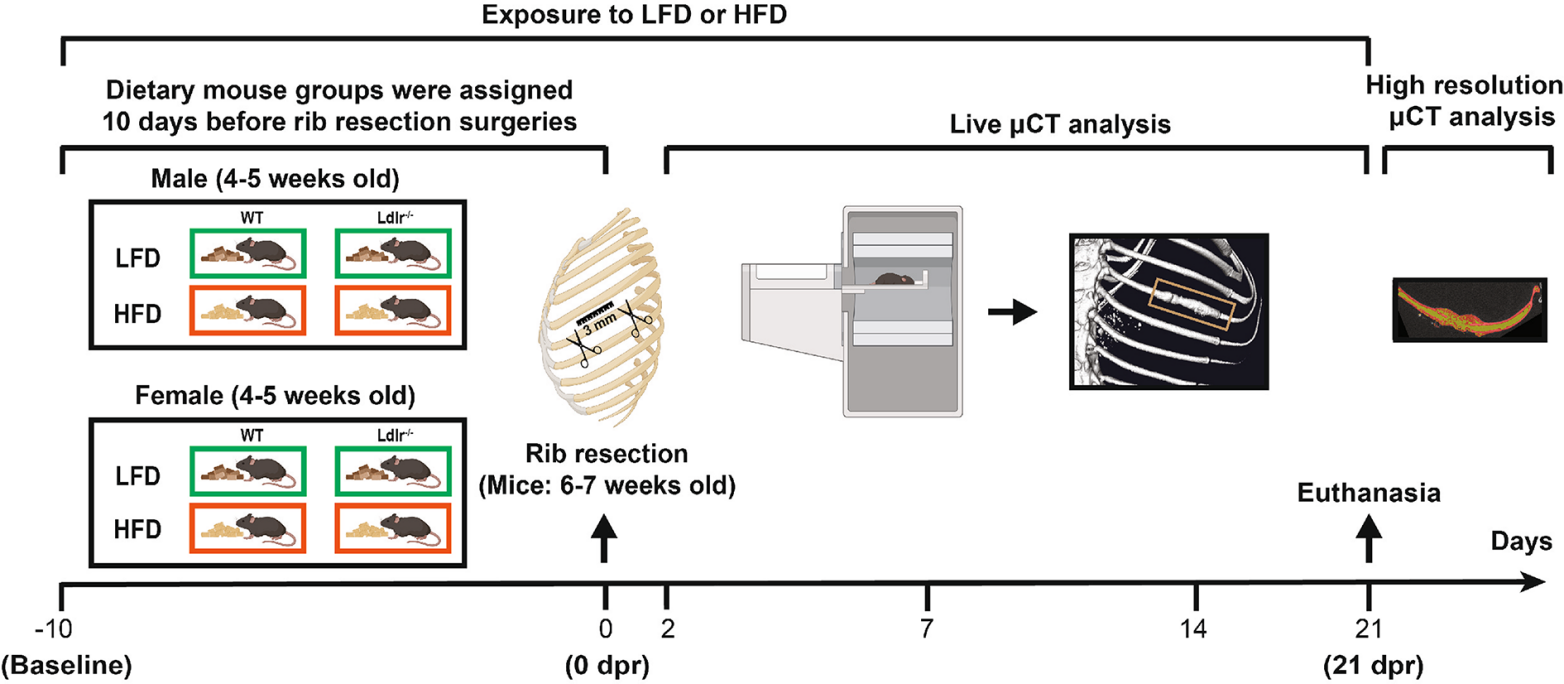
Schematic timeline of the mouse experimental setup. Young C57BL/6J mice (4–5 weeks old) from each strain (wild-type/WT and deficient for the low-density lipoprotein (LDL) receptor gene/Ldlr^−/−^) and sex were randomly assigned to different dietary groups 10 days before undergoing rib resection surgery (baseline). Animals were fed a low-fat diet (LFD) or high-fat diet (HFD) for a total of 31 days. Rib resection surgeries were performed 10 days after exposure to LFD or HFD (0 dpr) at 6–7 weeks of age and mice were monitored for any signs of bone regeneration by using live micro-computed tomography (µCT) data analysis at 2, 7, 14 and 21 days post-resection (dpr). At 21 dpr, all mice were euthanized, and ribs were dissected for subsequent analysis of ex vivo high resolution µCT data. The ruler shown at 0 dpr illustrates the length of the rib that is removed (3 mm) in each surgical procedure.

**Fig. 2:**
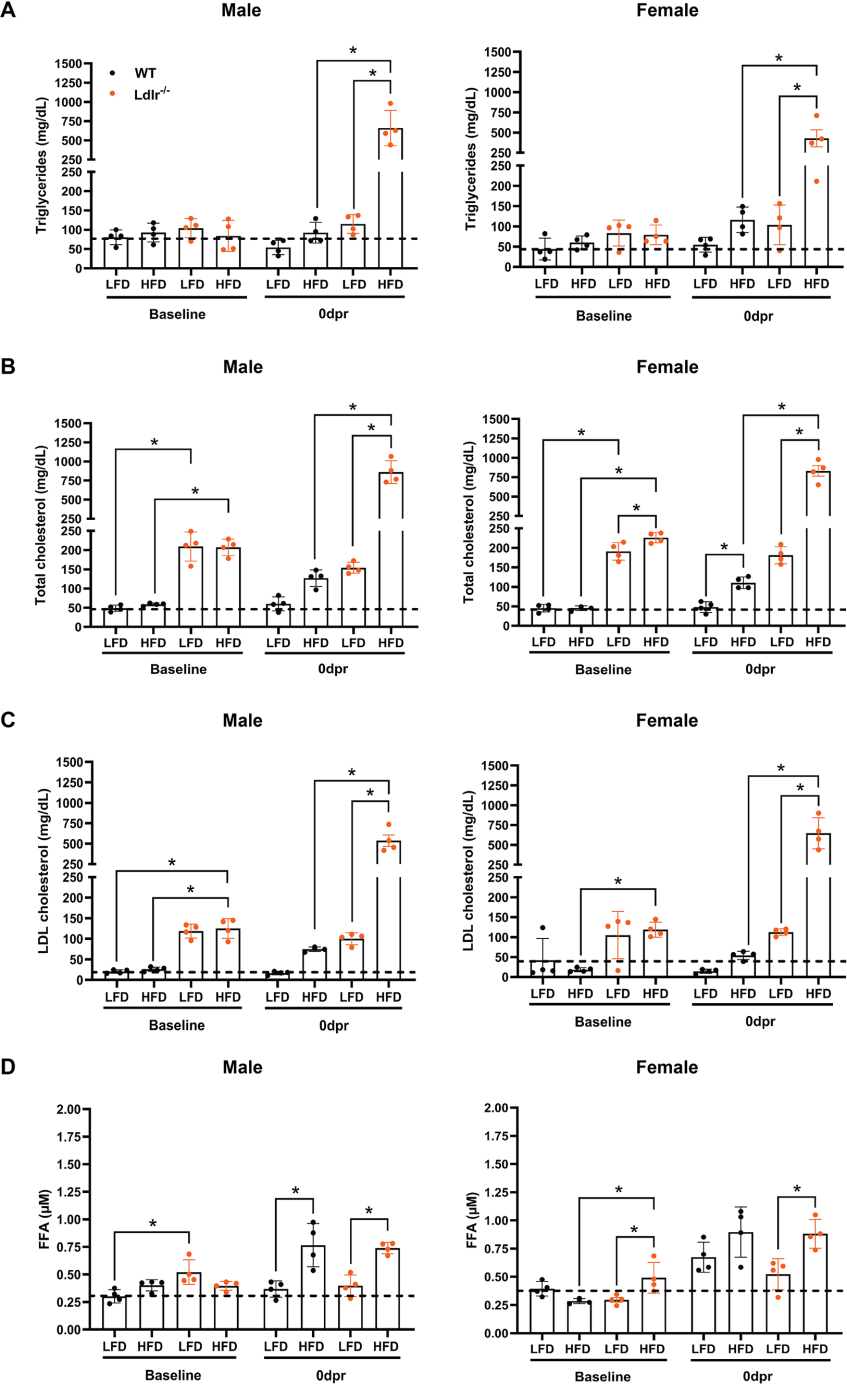
Systemic lipid profiles after HFD-induced hypercholesterolemia in Ldlr^−/−^ mice. Plasma concentrations of (**A**) triglycerides (mg/dL), (**B**) total cholesterol (mg/dL), (**C**) LDL cholesterol (low-density lipoprotein—mg/dL), and (**D**) FFA (Free fatty acids—µM—micromolar) in male and female mice with deletion of the low-density lipoprotein (LDL) receptor (Ldlr^−/−^) and age- and sex-matched wild-type (WT) control before (baseline) and 10 days after being exposed to a low-fat diet (LFD) or high-fat diet (HFD), which corresponds to the time point when the rib resection surgeries were performed (0 dpr). The dashed lines represent the overall mean value of the group of WT mice under LFD at baseline and were used as a reference in each graph for comparison with all other groups. Data are displayed as the mean ± SD (*n* = 4 in each group). Statistical analysis was performed using two-way analysis of variance (ANOVA) followed by Fisher’s LSD test. Significant differences between groups are expressed as *p < 0.05. dpr = days post-resection.

To test the potential relevance of lipid metabolism during bone regeneration, we utilized a previously established surgical rib resection procedure^6,7^ in an Ldlr^−/−^ (low-density lipoprotein, LDL, receptor) hypercholesterolemia mouse model. Specifically, to verify whether bone repair outcomes are distinct between the different sexes, young male, and female wild-type (WT/Ldlr^+/+^) and Ldlr^−/−^ C57BL/6J mice (4–5 weeks old) were fed a low-fat diet (LFD) or high-fat diet (HFD) for 10 days before rib resection and maintained on the same diet for 21 days post-resection (dpr), as shown in the timeline in Fig. 1. In our study, plasma lipid levels were measured in the peripheral blood of male and female WT and Ldlr^−/−^ mice before (baseline) and 10 days after being fed LFD or HFD (0 dpr), which corresponds to the time point when the rib resection surgeries were performed. At 0 dpr, the levels of triglycerides (Fig. 2A), total cholesterol (Fig. 2B), and LDL cholesterol (Fig. 2C) were significantly higher in both male and female Ldlr^−/−^ mice under HFD when compared to the corresponding WT mice under the same diet condition or Ldlr^−/−^ mice under LFD. The reference ranges for triglycerides and LDL cholesterol in the LFD-fed WT group at baseline were similar to values previously described for adult WT C57BL/6J mice (6–20 weeks old) in the absence of disease or abnormality^25^. Male and female Ldlr^−/−^ mice exhibited similar plasma free fatty acids (FFA) levels at 0 dpr and both had significantly higher values in the HFD groups when compared to their corresponding LFD controls (Fig. 2D). However, under LFD at 0 dpr, WT females exhibited considerably higher FFA values than WT males (Supplementary Fig. S1A), but among HFD-fed WT mice, plasma FFA levels were only significantly higher in male mice when compared to their corresponding LFD controls (Fig. 2D). In addition, under LFD, only WT females had a significant increase in FFA levels at 0 dpr, when compared to baseline (Supplementary Fig. S1B,C). These findings might help explain why different sexes are more or less susceptible to certain metabolic disorders. All female mice weighed significantly less than male mice when considering the same genotype and dietary condition at 0 dpr (Supplementary Fig. S2). Furthermore, no differences in weight gain were detected between groups, except for WT versus male Ldlr^−/−^ male mice under LFD and for female mice WT under LFD versus Ldlr^−/−^ under HFD.

### High-fat diet only affects BV in Ldlr^*−/−*^ male mice

**Fig. 3:**
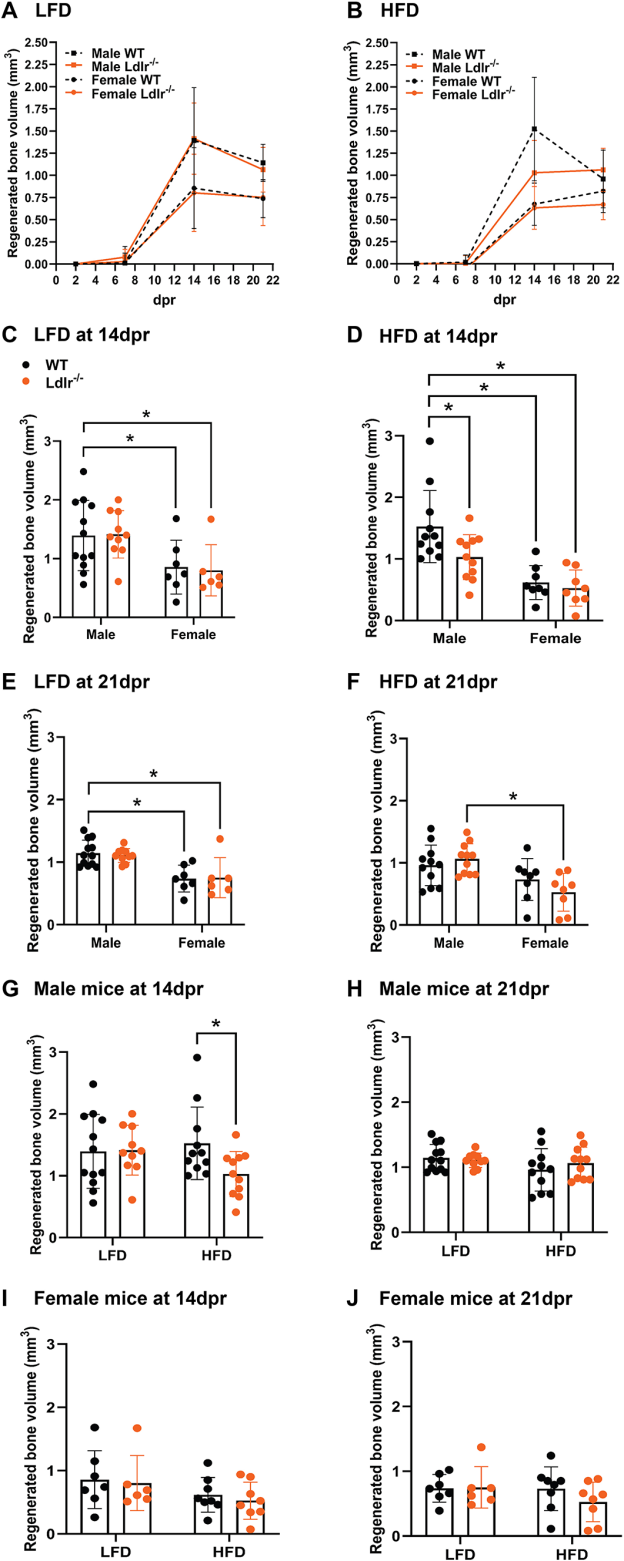
The effects of brief exposure to HFD are transient on bone regeneration in Ldlr^*−/−*^ male mice. Line graphs of bone callus volume (mm^3^) for each group of mice fed either (**A**) low-fat diet (LFD) or (**B**) high-fat diet (HFD) over time. Bar graphs showing callus bone volume (BV) comparison between groups of male and female mice at (**C**,**D**) 14 dpr and (**E**,**F**) 21 dpr under (**C**,**E**) LFD or (**D**,**F**) HFD. Bar graphs showing callus BV comparison between (**G-H**) groups of male mice with deletion of the low-density lipoprotein (LDL) receptor (Ldlr^−/−^) versus wild-type (WT) control and (**I**,**J**) female mice Ldlr^−/−^ versus WT under the same or different dietary condition (LFD or HFD). Data are displayed as the mean ± SD, considering WT male mice under LFD (*n* = 12), WT male mice under HFD (*n* = 11), Ldlr^*−/−*^ male mice under LFD (*n* = 10), Ldlr^*−/−*^ male mice under HFD (*n* = 11), WT female mice under LFD (*n* = 7), WT female mice under HFD (*n* = 8), Ldlr^*−/−*^ female mice under LFD (*n* = 6), and Ldlr^*−/−*^ female mice under HFD (*n* = 8). All BV measurements were obtained from live micro-computed tomography data analyzed using AnalyzePro 14.0 software. Statistical analysis was performed using two-way analysis of variance (ANOVA) followed by Fisher’s LSD test. Significant differences between groups are expressed as *p* < 0.05. dpr = days post-resection.

After mice underwent rib resection surgeries, bone regeneration was examined at 2, 7, 14, and 21 dpr using in vivo micro-computed tomography (µCT), as illustrated in Fig. 1. In this rib resection model, newly formed trabecular bone is already noticed at 14 dpr, and full repair can be expected within 1–2 months post-resection (28-56 dpr), as previously demonstrated^6,7^. Under LFD, WT and Ldlr^−/−^ mice did not show any significant differences in rib callus BV at any point in data analysis when considering animals of the same sex (Fig. 3A). However, under HFD, male Ldlr^−/−^ mice presented lower BV values when compared to male WT controls, while female mice showed similar bone regeneration capacity, regardless of genotype (Fig. 3B). The regenerated BV of WT males in Fig. 3B indeed decreased between 14 dpr and 21 dpr and recapitulated a similar result to when this same group was under LFD (Fig. 3A), suggesting that bone remodeling capacity was preserved regardless of being under HFD. It is noteworthy that the callus BV of male mice, WT or Ldlr^−/−^ mice, was generally larger than that of females with the corresponding genotypes at 14 dpr (Fig. 3C,D) or 21 dpr (Fig. 3E,F), regardless of the diet condition. Considering only the male group, Ldlr^−/−^ mice under HFD exhibited significantly reduced by 30% the regenerated BV compared to WT mice at 14 dpr, but not at 21 dpr (Fig. 3G,H). On the other hand, among females, there were no significant changes in callus BV between WT and Ldlr^−/−^ mice, either at 14 or 21 dpr, although there is a trend indicating that HFD appears to slightly reduce bone regeneration in female Ldlr^−/−^ mice when compared with female WT mice at 21 dpr (Fig. 3I,J). Taken together, these findings point to a transient impairment of rib regeneration in male mice under acute hyperlipidemic conditions. Conversely, females show a discernible reduction in relative regenerated BV compared to male mice, regardless of diet and genotype.

### The bone mineral density of regenerated bone is unaffected by diet or genotype

**Fig. 4:**
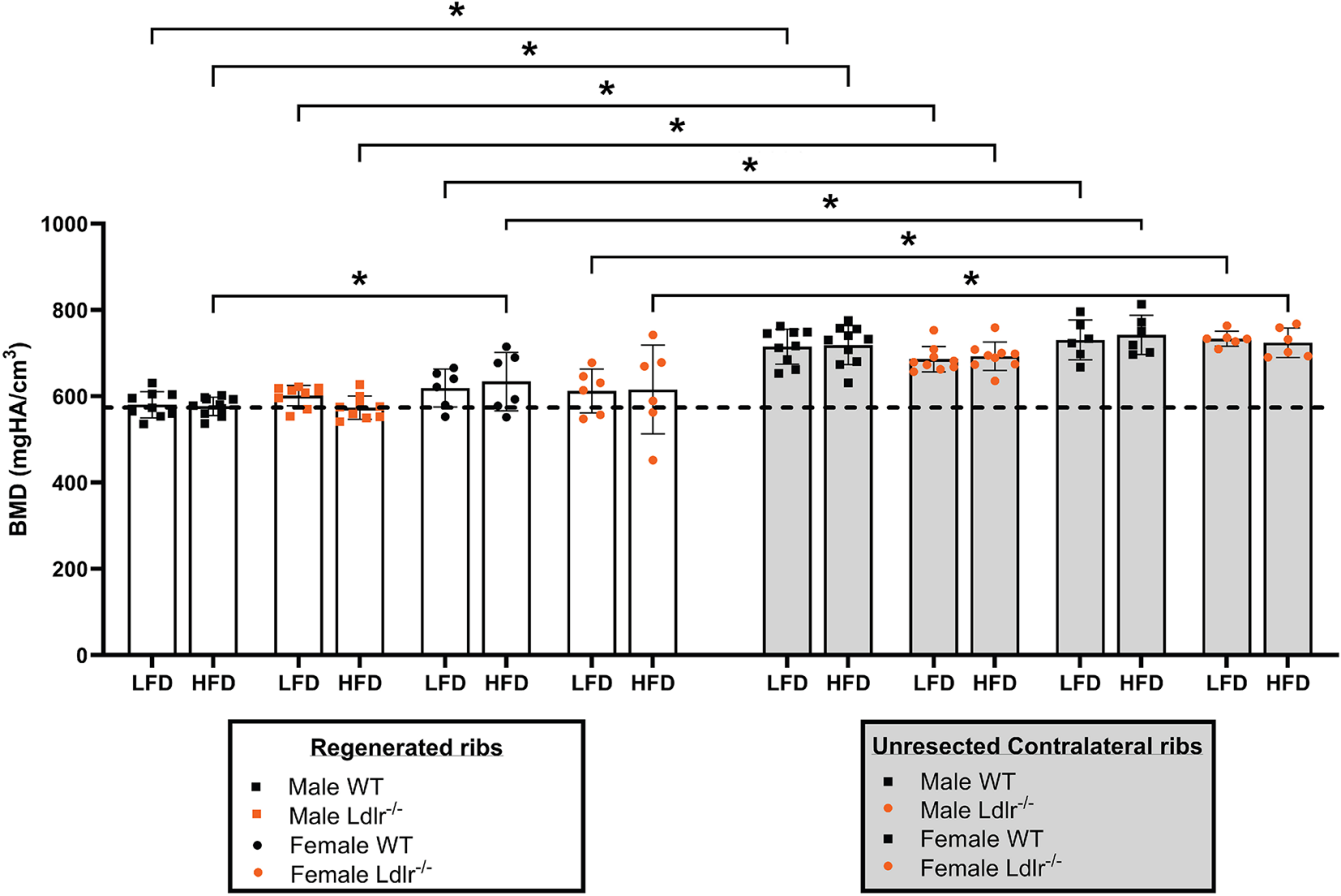
BMD remains unchanged between male and female mice under HFD. Bone mineral density (BMD—mg HA/cm^3^) of (**A**) regenerated rib callus and (**B**) its respective unresected contralateral rib in each mouse group at 21 dpr. The dashed line represents the overall mean value of the group of male wild-type (WT) mice under low-fat diet (LFD) and was used as a reference for comparison with all other groups. All BMD measurements were obtained from ex vivo high resolution micro-computed tomography data and analyzed using AnalyzePro 14.0 software. Data are displayed as the mean ± SD, considering WT male mice under LFD (*n* = 9), WT male mice under high-fat diet (HFD) (*n* = 10), Ldlr^−/−^ male mice under LFD (*n* = 9), Ldlr^−/−^ male mice under HFD (*n* = 9), WT female mice under LFD (*n* = 6), WT female mice under HFD (*n* = 6), Ldlr^−/−^ female mice under LFD (*n* = 6), and Ldlr^−/−^ female mice under HFD (*n* = 6). Statistical analysis was performed using two-way analysis of variance (ANOVA) followed by Fisher’s LSD test. Significant differences between groups are expressed as *p* < 0.05. dpr = days post-resection; high-fat diet (HFD).

Bone mineral density (BMD) was analyzed using ex vivo high-resolution µCT data from dissected regenerated ribs and their respective unresected contralateral ribs collected at 21 dpr. Among the regenerated ribs, there were no significant differences in BMD values between males and females, except for the rib calluses of WT male mice which are significantly less dense than those of WT female mice when both groups are under HFD (Fig. 4). On the other hand, all regenerated ribs showed significantly lower BMD values than the corresponding unresected contralateral ribs, regardless of sex, diet and diet, compatible with less mature bone during the early stages of trabecular bone formation. It is noteworthy that, in general, male mice tend to have a lower, more homogeneous distribution of BMD values, while female mice have a heterogeneous distribution of BMD values.

## Discussion

Normal cholesterol concentrations are essential for regulating the properties of cell membranes, such as receptor trafficking and signal transduction^26^. In the context of skeletal development, homeostasis and bone repair, cholesterol is most likely involved in the activation of osteochondrogenic pathways due to its relevance to the formation and homeostasis of lipid rafts, which are cholesterol-rich plasma membrane microdomains containing a variety of surface receptors, including those important for the function of osteoblast or osteoclasts^27–30^. Specifically, in mouse bone marrow-derived mesenchymal stem cells (MSCs), cholesterol loading has been shown to accelerate their differentiation by upregulating the transcription factors BMP2 (bone morphogenetic protein-2) and Runx2, which are critical for bone formation^31^. Conversely, osteoblast-like cells (MC3T3-E1) exposed to high concentrations of cholesterol exhibited reduced proliferation, differentiation, and expression of BMP2^32^. Consistent with in vitro data, rodents who underwent a HFD for at least three months exhibited decreased BMD, lower serum levels of alkaline phosphatase and osteocalcin^32^, and altered bone mechanical properties, mimicking the osteoporotic phenotype found in human patients^33^. Additionally, maternal hypercholesterolemia in pregnancy impairs embryonic skeletal development and Hedgehog signaling in offspring osteoblasts^34^. On the other hand, lipid-lowering drugs are known to promote osteogenic differentiation in vitro^35,36^, although clinical studies analyzing the efficacy of these agents on bone mass and fracture risk in human patients have not provided robust and consistent data^37,38^. Our current results are consistent with the notion that acute hyperlipidemia transiently affects the bone regeneration microenvironment, which makes conceivable the assumption that long-term exposition to HFD may cause long-standing chronic impairment of bone repair processes. However, although we observed that exposure to HFD for only 10 days significantly raised plasma lipid levels similarly in male and female Ldlr^−/−^ mice, the repair response to bone injury was exclusively altered in male Ldlr^−/−^ mice. Therefore, we speculate that female sex steroid hormones may prevent or at least reduce the adverse consequences of HFD on bone regeneration. Before menopause, endogenous estrogens are protective factors against atherosclerotic cardiovascular disease in women compared to men. On the other hand, the loss of estrogens after menopause is associated with dyslipidemia and an increased risk for osteoporosis^18–20,39^, while estrogen treatment increases BMD and prevents fractures in postmenopausal women^40^. Mechanistically, it was previously demonstrated that estrogens prevent bone loss by promoting mitochondrial apoptotic death of osteoclast progenitors^41^ and promoting bone formation by shifting the adipogenic and osteoblastic bipotential of MSCs to favor the osteoblast lineage^42^. Furthermore, intercellular communication through lipid-related pathways in the skeletal niche appears to play a role in bone repair. In support of this, Vi et al.^43^ reported that young macrophages produce factors, including the lipoprotein Lrp1, that promote osteoblast differentiation of bone marrow stromal cells from old mice, and that treatment of old mice with recombinant Lrp1 rejuvenated fracture repair. However, the potential involvement of lipid pathways in crosstalk between immune cells and skeletal stem and progenitor cells, directly influencing large-scale bone regeneration, should be investigated in further studies. Of note, a previous study showed that female mice, but not male mice, are protected from HFD-induced metabolic changes such as glucose intolerance and hyperinsulinemia, likely due to the ability to expand the anti-inflammatory Treg (regulatory T cell) population in intra-abdominal adipose tissue^44^. Therefore, regulation of immune cell activity by steroid hormones^45^ may modulate bone regeneration outcomes in individuals with hyperlipidemia. To our knowledge, the present study is the first study investigating differences in the repair of large defects bone between male and female mice using a rib resection model. Although sex differences are evident in terms of bone regeneration capacity, repaired BV in female mice is lower than in male mice, regardless of diet or genotype. These results are corroborated by a previous study carried out with C57BL/6J mice and showing that males have faster healing than females in a fracture model^46^. Furthermore, a recent study demonstrated sex-dependent differences in the inflammatory response during the repair of fractures and smaller calluses formed in female mice than in male counterparts, regardless of age^47^. It has also been suggested that sex-related differences in repairing bone are potentially a result of weight-dependent mechanical stimulation differences between the sexes. However, the differential activation of osteogenic pathways and its correlation with inflammatory response and hormonal regulation remains to be investigated in future studies. In addition, as the resected bone in this study is not weight bearing, the effect of individual weight is likely not a contributing factor.

Together, our findings show that, even with a comparable hyperlipidemia, sex differences were noted during bone regeneration in HFD-fed mice, but changes were transient. Thus, it is necessary to understand the complexity of the effects of sex hormones on the bone regeneration microenvironment in hyperlipidemic settings. Although the current study shows promising findings, more studies should be done to further confirm these results since the sample size in each group at the start of the study may be different from the n numbers in the analysis given the complexity of the surgical procedures which impact survival.

## Electronic supplementary material

Below is the link to the electronic supplementary material.

Supplementary Material 1

## Data Availability

All data needed to support the findings of this study are available within the manuscript and its supplementary information files.
