# Supplementary material for: Acute hyperlipidemia has transient effects on large-scale bone regeneration in male mice
Source: Sci Rep. 2024 Oct 27;14:25610. doi: 10.1038/s41598-024-76992-9 (PMC11514207; doi:10.1038/s41598-024-76992-9)
Supplement: Supplementary file 1 — Supplementary Material 1 [file 41598_2024_76992_MOESM1_ESM.docx]

**Supplementary Information**

**Transient severe hyperlipidemia has mild effects on large-scale bone regeneration in male mice.**

Authors: Luciana Yamamoto de Almeida^1^*, Catharine Dietrich^1^, Olivier Duverger^1^, Janice Lee^1^*

Affiliations:

1. Craniofacial Anomalies and Regeneration Section, National Institute of Dental and Craniofacial Research (NIDCR), National Institutes of Health (NIH), Bethesda, MD 20892, USA.

**Supplementary Fig. S1**

**
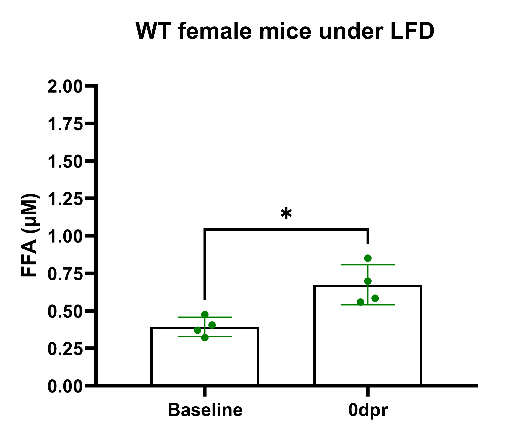
**
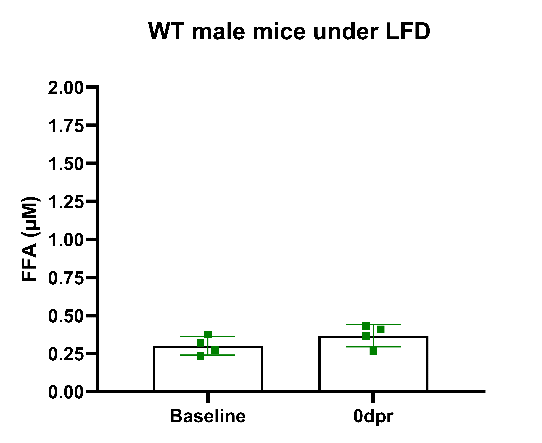
**
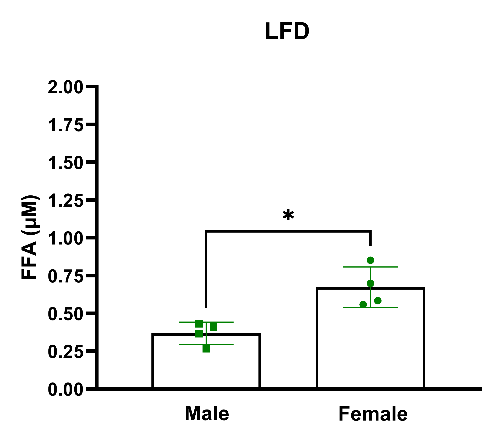
**

**C**

**A**

**B**

**Supplementary Figure S1.** Plasma free fatty acid (FFA) levels in male and female wild-type (WT) mice under low-fat diet (LFD). Plasma FFA levels (μM – micromolar) were measured in the peripheral blood of male and female WT mice before (baseline) and 10 days after from being fed a low-fat diet (LFD), which corresponds to the time point when the rib resection surgeries were performed (0dpr). Bar graphs showing plasma FFA levels comparison between (**A**) groups of male and female mice at 0dpr under LFD, (**B**) WT male mice at baseline versus 0dpr under LFD, and (**C**) WT female mice at baseline versus 0dpr under LFD. Data are displayed as the mean ± SD. Statistical analysis was performed using Mann-Whitney test; *p < 0.05 (n= 4 animals/group). dpr = days post-resection.

**Supplementary Fig. S2**


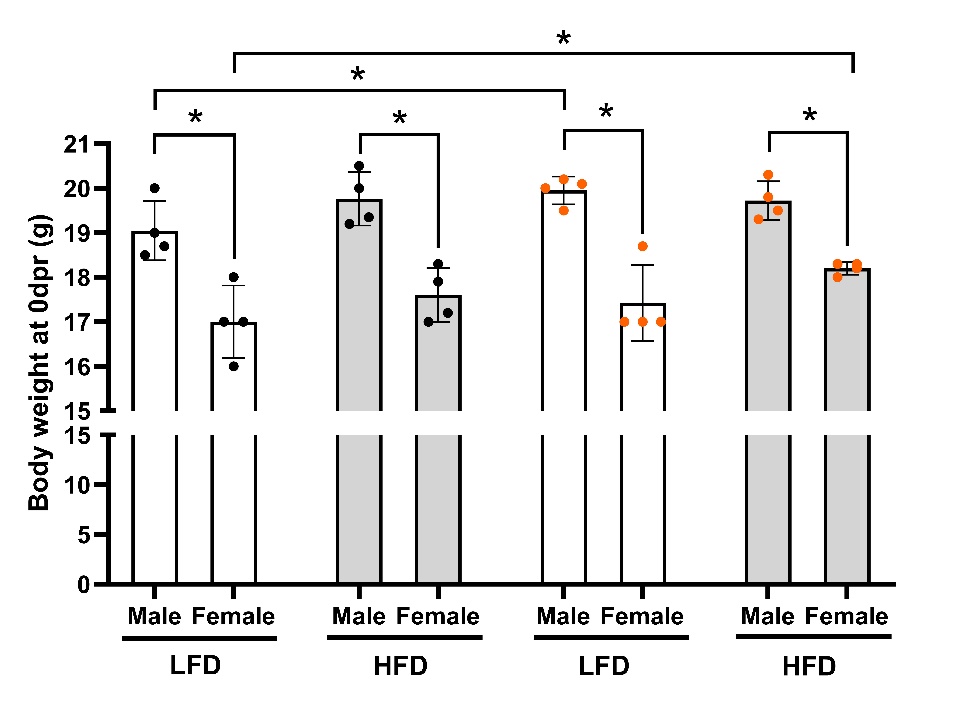


**Supplementary Figure S2.** Body weight differences between male and female mice under LFD or HFD. The chart shows the change in body weight (g – grams) of each group of mice (6-7 weeks old) at 0dpr. Data are displayed as the mean ± SD. Statistical analysis was performed using two-way analysis of variance (ANOVA) followed by Fisher's LSD test. Significant differences between groups are expressed as *p < 0.05 (n= 4 animals/group). dpr = days post-resection; low-fat diet (LFD); high-fat diet (HFD); wild-type mice (WT); mice with homozygous deletion of the low-density lipoprotein (LDL) receptor (Ldlr^−/−^).
